# CardioFit: a WebGL-based tool for fast and efficient parametrization of cardiac action potential models to fit user-provided data

**DOI:** 10.1098/rsos.250048

**Published:** 2025-08-27

**Authors:** Darby I. Cairns, Maxfield Roth Comstock, Flavio H. Fenton, Elizabeth M. Cherry

**Affiliations:** ^1^ School of Computational Science and Engineering, Georgia Institute of Technology, Atlanta, GA, USA; ^2^ Department of Physics, Georgia Institute of Technology, Atlanta, GA, USA

**Keywords:** cardiac modelling, parameter fitting, particle swarm optimization, identifiability

## Abstract

Cardiac action potential models allow examination of a variety of cardiac dynamics, including how behaviour may change under specific interventions. To study a specific scenario, including patient-specific cases, model parameter sets must be found that accurately reproduce the dynamics of interest. To facilitate this complex and time-consuming process, we present CardioFit, an interactive browser-based tool that uses the particle swarm optimization (PSO) algorithm implemented in JavaScript and takes advantage of the WebGL API for hardware acceleration. Our tool allows rapid customization and can find low-error fittings to user-provided voltage time series or action potential duration data from multiple cycle lengths in a few iterations (10–32), corresponding to a runtime of a few seconds on most machines. Additionally, our tool focuses on ease of use and flexibility, providing a webpage interface that allows users to select a subset of parameters to fit, set the range of values each parameter is allowed to assume, and control the PSO algorithm hyperparameters. We demonstrate our tool’s utility by fitting a variety of models to different datasets, showing how convergence is affected by model choice, dataset properties and PSO algorithmic settings, and explaining new insights gained about the physiological and dynamical roles of the model parameters.

## 1. Background

Mathematical models are widely used to predict and analyse the electrical behaviour of cardiac cells and tissue. Although solving the differential equations that underlie most modern models of cardiac action potentials [1] is a challenging numerical task [2], due to the often large number of variables required to describe them as well as the large number of cells required for two- and three-dimensional simulations, software like Chaste [3], OpenCarp [4] and a set of WebGL programs [5,6] have been developed to simplify obtaining model solutions. However, even relatively simple cardiac models include many parameters whose values can be modified to represent cells and tissue under different conditions and to accommodate the inherent variability between individuals [7]. Because these parameters have complex, interacting effects on the model output, finding a set of parameters for a model that accurately captures the relevant behaviour of data recorded from experiments or from a different model is challenging.

A variety of approaches have been used to tackle the problem of finding appropriate parameter values, including simulated annealing [8], least squares [9] and gradient-based approaches [10], and regression methods [11,12]. Genetic algorithms, which maintain a pool of candidate solutions (parametrizations) that are improved using biologically inspired crossover, selection and mutation transformations, also have been widely used [13–17]. Particle swarm optimization (PSO) similarly maintains a pool of candidate solutions, but rather than evolving the individuals according to rules inspired by genetics, the candidates move in random degrees towards the best solutions that have been identified in previous iterations [18–20]; it has also been combined with a quadratic optimization algorithm in a hybrid approach to improve convergence [21]. Techniques like data assimilation [22,23] also have been used to estimate parameter values along with the system state. As an alternative to more traditional optimization methods, Bayesian approaches have been used to estimate not a single set of parameter values but a posterior probability distribution—given a model output, how likely a particular model parametrization is—using a variety of algorithms, such as Markov chain Monte Carlo [24], approximate Bayesian computation [25,26], Bayesian active learning [27], Bayesian history matching [28] and Hamiltonian Monte Carlo [29,30]. Implementations for many of these approaches to fitting cardiac model parameters may require extensive programming, so that the selection of a technique for a particular application may depend on prior experience and preferences.

In the present study, we present CardioFit, a tool to find cardiac model parametrizations that is fast, flexible, easy to use, and applicable in many settings without requiring detailed understanding of the model parameters or optimization algorithm. To promote computational efficiency, we utilize the parallelism provided by graphics hardware on modern computers, which influences our choice of optimization approach towards one that can benefit from this architecture. Towards this end, CardioFit uses PSO to optimize parameter values for several cardiac models to match user-provided datasets. PSO requires updating a pool of candidate solutions over multiple iterations, with each candidate solution at each iteration requiring a full solution of the model to be compared with the data. Due to its limited communication needs, the PSO algorithm is well suited to be executed on thousands of cores in parallel. Consequently, CardioFit can implement PSO with a large candidate pool (up to tens of thousands) and often can identify a low-error parameter fitting within a few dozen iterations in seconds, even on modest hardware, without sacrificing accuracy. As CardioFit uses the WebGL API for JavaScript and is implemented as a webpage, no machine-specific manual compilation or installation is necessary to optimize performance. The only setup required is visiting a website hosting the program or downloading the code and opening it in a Web browser. The source code for the CardioFit program is publicly available on GitHub,^1^ and the program can be accessed directly online.^2^

Previously, we have shown that this approach is capable of fitting a cardiac action potential (AP) model to data taken from explanted human hearts with Brugada syndrome [31] and to data generated from complex cardiac models [20]. In this work, we present a detailed description of CardioFit’s implementation and quantify its effectiveness. We find that CardioFit can fit datasets taken from biophysically complex models to several phenomenological and minimal cardiac models, accurately capturing key properties of the original data, such as AP duration (APD), alternans and AP morphology. We demonstrate similarly accurate fittings for a variety of experimental datasets, with as many model parameter values being fitted simultaneously as desired. We also show that with available graphics hardware, a large number of candidate solutions can be used to find appropriate model parametrizations with just tens of PSO iterations. In addition, the fitting process leads to additional insights into the roles of parameters in cardiac models as well as identifiability.

## 2. Methods

In this section, we provide information about the cardiac models and their simulation, the optimization algorithm and the datasets considered as well as CardioFit’s design and implementation. Additional details are available in the electronic supplementary material.

### 2.1. Cardiac action potential models

The two- to four-variable cardiac models available in CardioFit describe the change in a dimensionless voltage variable u over time:


(2.1) $$\frac{du}{dt}=I_{\text{tot}}+I_{\text{stim}},$$

where $I_{\text{tot}}$ is a function of u and one or more variables that evolve in time according to the specific model and $I_{\text{stim}}$ is an external stimulus current. The models currently included in CardioFit are the modified FitzHugh–Nagumo (MFHN) [32], Mitchell–Schaeffer (MS) [33], modified Mitchell–Schaeffer (MMS) [34], Fenton–Karma [35], Bueno–Orovio–Cherry–Fenton (BOCF) [36] and Brugada BOCF (BBOCF) models [37]. Detailed equations for each model are supplied in the electronic supplementary material. Models are integrated using the forward Euler method with a time step of 0.02 ms.

Two types of stimulus current are available to the user. The default option is a square stimulus, which is set to a non-zero constant for a fixed amount of time and is zero otherwise. The default magnitude of the stimulus is 0.2 ms^−1^ and the default duration is 2 ms. These values can be changed in the user interface, as described in electronic supplementary material, §S1.3.1. Additionally, a biphasic stimulus designed to mimic the upstroke-inducing current experienced via diffusive coupling is available. Details are given in the electronic supplementary material.

### 2.2. Experimental and model-derived datasets

To demonstrate the versatility of CardioFit, we show fittings to both experimental and model-derived data. Our experimental datasets include recordings from four different datasets: fish, frog, canine endocardial and human. The fish, frog and canine datasets are from microelectrode recordings from a single ventricular cell in tissue paced at one or more constant periods, or cycle lengths (CLs). The human ventricular dataset consists of time series taken from single pixels of optical-mapping recordings of an explanted human heart displaying characteristics of Brugada syndrome. The time resolution of all experimental datasets is 1 ms. Datasets used were recorded with CLs of 500 ms, 400 ms, 320 ms and 260 ms for canine data, 300 ms for fish data, 800 ms for frog data and 1000 ms for human data.

Model-derived data were generated from any of the models available within CardioFit and from more complex cardiac action potential models, specifically, the Fox *et al.* model [38] and the ten Tusscher *et al.* model [39]. These models were integrated using the Rush–Larsen method for gating variables and forward Euler for other variables with a time step of 0.005 ms, and the corresponding datasets all had a time resolution of 1 ms. Calcium concentrations in the ten Tusscher *et al.* model were updated using the analytical approach described in appendix 1 of [40]. Pacing CLs of 500 ms, 400 ms and 300 ms were used for each of the MS, FK and BOCF models. For the Fox *et al.* model dataset, pacing CLs of 500 ms, 400 ms, 300 ms and 225 ms were used. Data with CLs of 500 ms, 400 ms, 350 ms and 310 ms were used from the ten Tusscher *et al.* model. In all cases, the model was paced until a steady state was reached for the given CL before recording the data.

The input data used for the PSO fits in this paper are available in the source code repository.^3^

### 2.3. Particle swarm optimization algorithm and CardioFit implementation

To identify appropriate parameter values that fit any of the models listed in §2.1 to data, CardioFit uses PSO, a derivative-free optimization method that does not require specific assumptions about the problem being optimized. It maintains a pool of candidate solutions (‘particles’) that are iteratively updated, using knowledge from the previous particle states, until a fixed number of iterations is completed (PSO can also be implemented to terminate when some threshold criterion is achieved, or the particles converge to a stable result). Each particle has a position and velocity with d dimensions, where d is the number of parameters of the model. The position $p_{i}$ of particle i is a vector consisting of the current values of each of the parameters for that particle, and the velocity is used to determine changes in the position according to information acquired from the group of particles. Information used to update the velocity of a particle i includes the best position it has ever achieved, $b_{i}$, and the best position ever found by any particle, b.

For fitting cardiac models to voltage data, CardioFit uses the sum of squared error between the normalized data and the voltage of the model as the fitness metric. The program automatically aligns the first upstroke of the model output and the data to perform the comparison. CardioFit also can be used to fit the model to APD values in addition to or instead of a full voltage time series, in which case the squared APD error is used. When both types of error are considered or multiple datasets are provided, an additional parameter can be adjusted to balance their relative weights. The error is automatically weighted by the length of the data file so that longer datasets (corresponding to longer CLs) do not necessarily tend towards higher error. For a given dataset, the quality of a particular particle is determined by the value of the fitness function evaluated using its parameter values.

The position of each particle is initialized from a uniform random distribution over the valid range of values for each parameter. The position and velocity of each particle are updated at each iteration n according to


(2.2) $$\begin{array}{rl} v_{i}^{n+1} & =\begin{array}{rl} \chi [v_{i}^{n}+ & u(0,\phi_{1})⊙\left(b_{i}^{n}-p_{i}^{n}\right)\\ + & u(0,\phi_{2})⊙\left(b^{n}-p_{i}^{n}\right)], \end{array}\\ p_{i}^{n+1} & =p_{i}^{n}+\gamma v_{i}^{n+1}, \end{array}$$

where χ is a constriction coefficient used to promote convergence in the algorithm [41], γ is the learning rate that governs how aggressively particle positions are changed, u(a,b) is a d-dimensional vector of uniformly distributed random numbers in the range [a,b), and ⊙ is the Hadamard product. Each parameter is restricted to a range of possible values; if an updated particle contains any parameter value outside its range, that value is reset to a random position within the nearest three-quarters of the range. The values of $\phi_{1}$, $\phi_{2}$ and χ are hyperparameters that may be adjusted as desired using the user interface as described in electronic supplementary material, §S1.3.1. The results presented here use CardioFit’s default values of $\phi_{1}=\phi_{2}=2.05$ and $\chi =2/(\phi -2+\sqrt{\phi^{2}-4\phi })\approx 0.73$ with $\phi =\phi_{1}+\phi_{2}$ (following [21]), as well as γ=0.05, which was determined empirically.

The PSO algorithm relies on a pool of particles to explore parameter space and find viable solutions. Because the evaluation of each particle requires finding the solution to the model and comparing the result with each value of the input data to fit, each iteration of the algorithm incurs a computational effort proportional to the number of particles. However, these model runs require no interaction between particles, and therefore may be performed in parallel with no modification to the algorithm. Other steps, such as the particle update step, may also be performed in parallel to further increase the speed of the program and avoid unnecessary data transfer. More details are available in electronic supplementary material, §S1.4.

### 2.4. CardioFit interface

**Figure 1.**
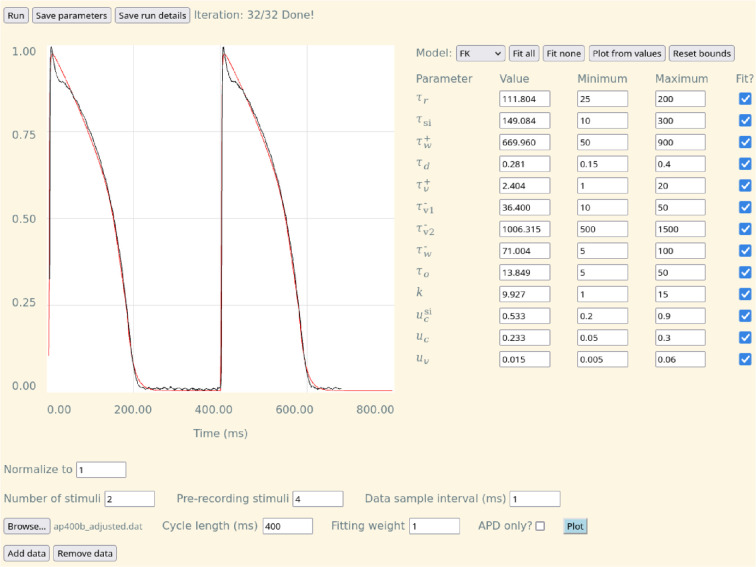
User interface of CardioFit. Top row: buttons to initiate and save the results of CardioFit runs and a progress display for the iterations. Left: fit (red) to the data (black) for the selected cycle length. Right: interface for choosing the model, selecting the parameters to fit, setting the bounds, and viewing the fit parameter values. Bottom: interface for adding datasets to fit.

**Figure 2.**
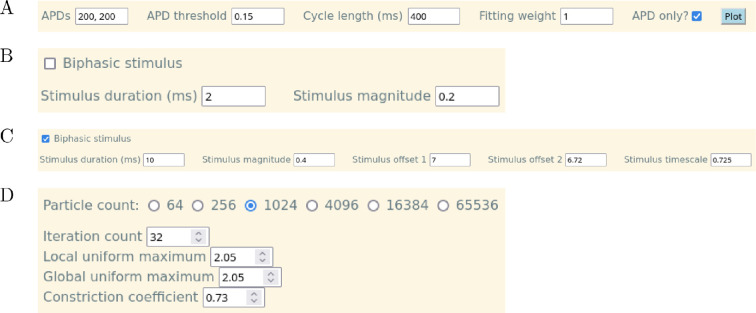
Detailed view of several elements of the CardioFit user interface. (A) APD data entry interface for CardioFit. (B) Square stimulus parameters for CardioFit. (C) Biphasic stimulus parameters for CardioFit. (D) The interface to configure the PSO algorithm hyperparameters in CardioFit.

The user interface of CardioFit provides access to the key features of our PSO implementation without requiring knowledge of the underlying implementation. The following sections contain a brief description of the provided behaviour and intended workflow for the program. Screenshots of the main user interface and additional interface elements of CardioFit are shown in figures 1 and 2 for reference. More details are provided in the electronic supplementary material. Documentation for the program is also available in the source repository in the README page.^4^

#### 2.4.1. Specifying data to fit

Fitting a model to a dataset in CardioFit requires adding the data and setting a few options; see figure 2A. CardioFit expects newline-delimited files containing a sequence of voltage values. The data are normalized by the program to have a minimum value of zero and a maximum value matching the number entered in the ‘Normalize to’ entry field. If the dataset is already normalized to the desired range, this behaviour can be bypassed by setting the value of the ‘Normalize to’ field to 0. Default values for the ‘Normalize to’ field vary by model and are given in electronic supplementary material, §S1.1; these values are used in the results presented here unless noted otherwise. Note that within the figures all results have been normalized to the same 0−1 scale, even if a normalization constant other than 1 was used for a fitting.

Aside from normalization, other pre-processing steps outside of CardioFit may be necessary. For instance, high-resolution data should be downsampled to a sample interval of around 1 ms, as large data files may not fit in GPU memory on systems without powerful dedicated graphics hardware. When comparing the model output with a candidate parametrization to the provided data, the model is first run for a user-specified number of stimuli, set in the ‘Pre-recording stimuli’ field, to reduce transient effects of the initial conditions. After these stimuli have been applied, the simulation continues by applying the number of stimuli specified as the ‘Number of stimuli’ field, and the results are compared with the data.

To add a single data file, the ‘Browse’ button above the ‘Add data’ button is clicked and the file is selected. Along with this file, the pacing period is entered in the ‘Cycle length’ field to the right. Additional files with different pacing periods can be added by clicking the ‘Add data’ button to add additional rows for data entry. These rows may be removed by clicking the ‘Remove data’ button. The ‘Fitting weight’ field allows the different files to be given greater or lesser relative priority in the PSO fitness function when multiple files are used. When the total error for a particular particle is computed, the error from each dataset is multiplied by its fitting weight, and then all the components are summed. It is recommended to leave the fitting weights at the default value of 1 for initial attempts and adjust them to fine-tune the behaviour of subsequent fits. For example, data from a particular cycle length could be given extra weight in an attempt to fit a bifurcation or alternans details. Additionally, the number of stimuli to apply, number of pre-recording stimuli to be applied before fitting, and sample interval of the data must be the same for all data files and entered above the data rows. Larger numbers of stimuli and smaller sample intervals will increase the time required for the program to run.

The ‘Plot’ button in each data row can be used after selecting a file to plot the corresponding raw data as a time series. After running CardioFit, the functionality of this button changes to plot both the data and the model fit.

If only APD data are available, then the ‘APD only’ checkbox gives the option to fit to APDs. When the checkbox is selected, the data row changes to contain the entries shown in figure 2A. Instead of selecting a file, the APD values are entered directly in the ‘APDs’ field as a comma-separated list of numbers. The number of APDs entered in the ‘APDs’ field should match the value in the ‘Number of stimuli’ field above, so that one correct value is provided for comparison with each simulated action potential. The ‘APD threshold’ corresponds to the normalized voltage value at which the APD is measured: e.g. ${\text{APD}}_{90}$ for data normalized to 1 would be specified by entering 0.1.

It is possible to use CardioFit to fit voltage and APD data simultaneously, in which case the ‘Fitting weight’ field becomes relevant to tune the relative importance of the datasets. To fit both voltage and APD data, multiple data rows must be added as described above, with separate rows representing the voltage data and the APD data.

#### 2.4.2. Choosing a model and parameter ranges

The fields to the right of the graph window are used to select the model whose parameters will be fitted and to adjust model parameter settings. Once a model is selected by clicking the ‘Model’ drop-down menu and choosing one of the options, the other fields automatically change to display the relevant parameters and default values. Each row beneath the model name contains, from left to right, the parameter name, the value of that parameter (initially empty), the minimum allowable value of the parameter for the fitting, the maximum allowable value of the parameter for the fitting, and a checkbox indicating whether or not that parameter is to be fitted. The ‘Fit all’ and ‘Fit none’ buttons provide convenient shortcuts to (respectively) check or uncheck all of these boxes. If a parameter is not set to be fitted, a value must be provided in the ‘Value’ column. For all other parameters, the range of values explored by PSO can be adjusted by changing the maximum and minimum values. All results presented here use the default parameter bounds for each model (see electronic supplementary material, tables S1–S6) unless stated otherwise.

To generate a fit using CardioFit, click the ‘Run’ button at the top of the page. The current iteration number is displayed while the program runs. Once the program has completed, the result of the fit compared with the first data file is plotted in the graph window, as shown in figure 1, with the model fit in red and the data in black. A comparison of the output fit for a different input data file may be viewed by clicking the ‘Plot’ button next to the file of interest. A plot of the global best error (vertical axis) as the number of iterations increases (horizontal axis), as shown in electronic supplementary material, figure S2, is plotted beneath the PSO hyperparameters.

#### 2.4.3. Interpreting and saving the results

After the iterations are completed, the best-fit value resulting from the fitting is displayed in the ‘Value’ column for each parameter. Note that displayed values are rounded to three significant digits; for full precision, the parameter values can be saved to a file by clicking the ‘Save parameters’ button. The parameter values and other additional information about the CardioFit run, such as the PSO hyperparameter values, can be saved to a file by clicking the ‘Save run details’ button. Because CardioFit runs in a Web browser, the file is treated as a download; it may appear in the ‘Downloads’ folder, or a prompt to select a save location may appear, depending on the browser settings.

### 2.5. Statistical analysis

For comparisons of parameter values obtained across multiple fittings of a given model to different datasets, we used the standard *t*‐test. Because of the large number of model parameters and dataset comparisons, we used a small *p-*value of 0.001 to determine significance, but we also identified cases with *p*-values between 0.001 and 0.01. For all comparison cases, we used 20 runs with 4096 particles and 32 iterations.

## 3. Results

In this section, we demonstrate the effectiveness of CardioFit in fitting both model-generated data and experimental data and highlight how CardioFit can lead to insights about the roles of the parameters in the models being fit. We also analyse CardioFit’s convergence and scaling properties.

### 3.1. Fitting to data from the same model

**Figure 3.**
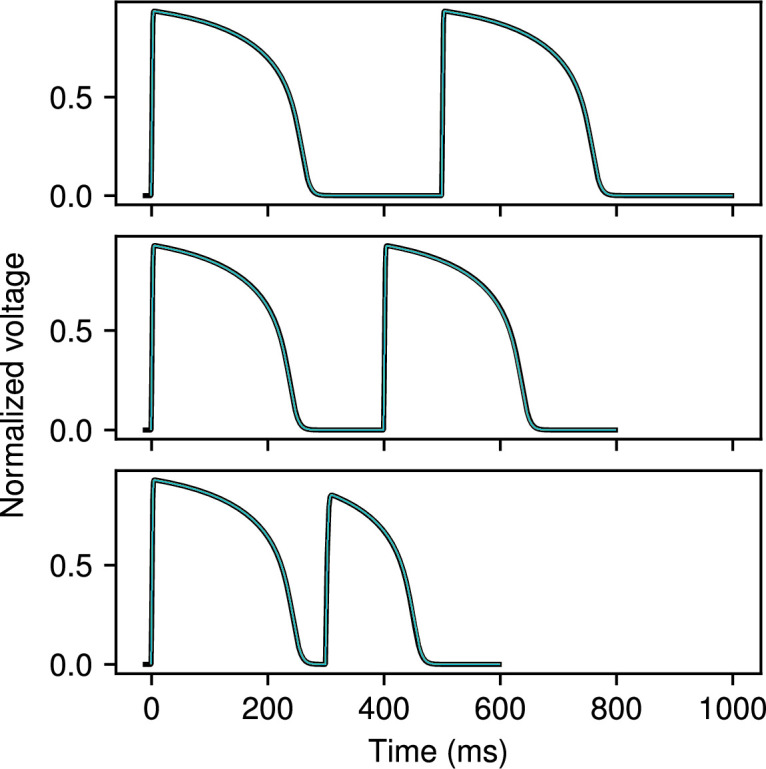
MS model fit to voltage data obtained from the same model. Cycle lengths of 500 ms, 400 ms and 300 ms were fitted simultaneously to the two action potentials shown. Reference data are plotted in black; the results of 20 separate fits using CardioFit are plotted in various colours. Fits were generated using 4096 particles, 100 iterations and four pre-recording stimuli in all cases.

**Figure 4.**
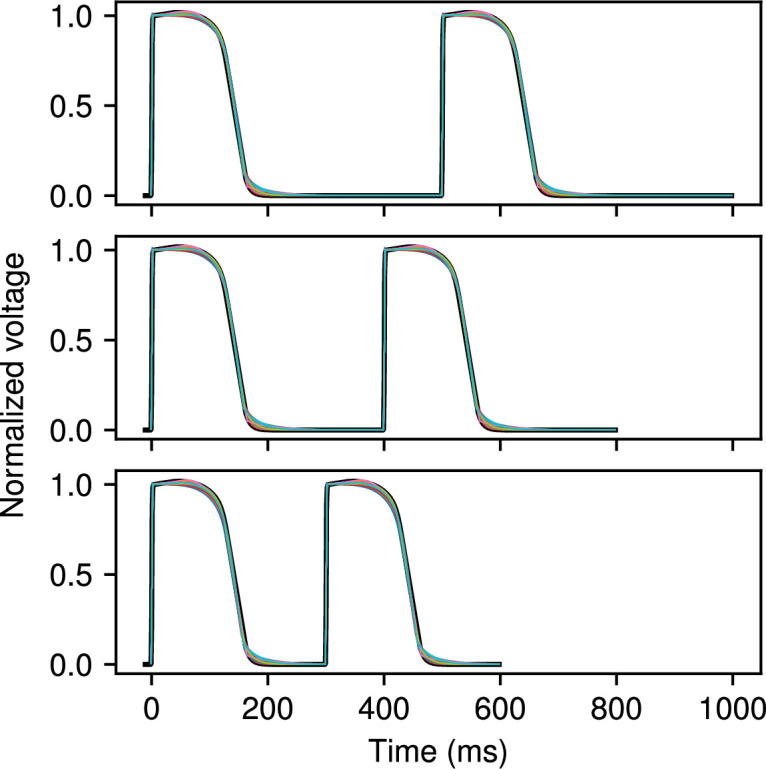
FK model fit to voltage data obtained from the same model. Cycle lengths of 500 ms, 400 ms and 300 ms were fitted simultaneously to the two action potentials shown. Reference data are plotted in black; the results of 20 separate fits using CardioFit are plotted in various colours. Fits were generated using 4096 particles, 100 iterations and four pre-recording stimuli in all cases.

**Figure 5.**
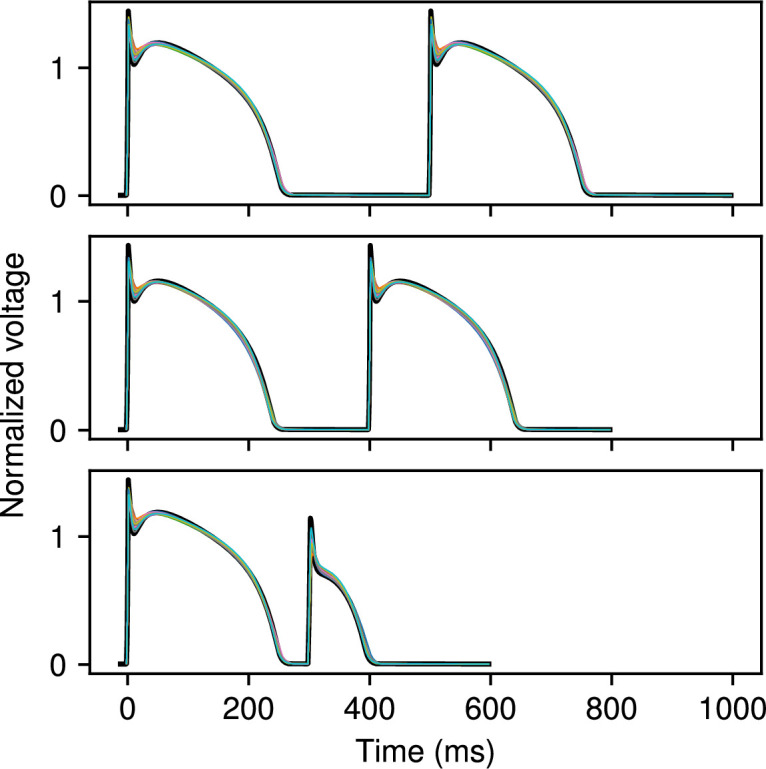
BOCF model fit to voltage data obtained from the same model. Cycle lengths of 500 ms, 400 ms and 300 ms were fitted simultaneously to the two action potentials shown. Reference data are plotted in black; the results of 20 separate fits using CardioFit are plotted in various colours. Fits were generated using 4096 particles, 100 iterations and four pre-recording stimuli in all cases.

A first test of CardioFit is fitting a model to data derived from the same model, which allows us not only to evaluate the quality of the fit (how close the voltage and/or APD values are to the user-provided data), but also to assess how reliably the known parameters used to create the model-generated dataset can be identified from the data. Due to randomness in the particle initialization and velocity updates, combined with limitations in parameter identifiability from the model structure or dataset(s) being fitted (or both), repeated fitting attempts of a model to data generated from the same model may result in different parameter values that result in slightly different voltage traces. We tested this behaviour with the MS, FK and BOCF models, which were fitted to three cycle lengths simultaneously. Figures 3–5 show the data from the true model parametrizations in black and the result of 20 different fittings overlaid in colour for the three models. Because the MS model has only five parameters, the 20 fittings produced results that were difficult to distinguish visibly, as shown in figure 3. For the FK model, variability in the fitted voltage values obtained occurred during the AP plateau and towards the end of repolarization, as can be seen in figure 4. Figure 5 shows that for the BOCF model, differences were evident primarily in the height of the upstroke as well as during the initial repolarization and plateau.

**Figure 6.**
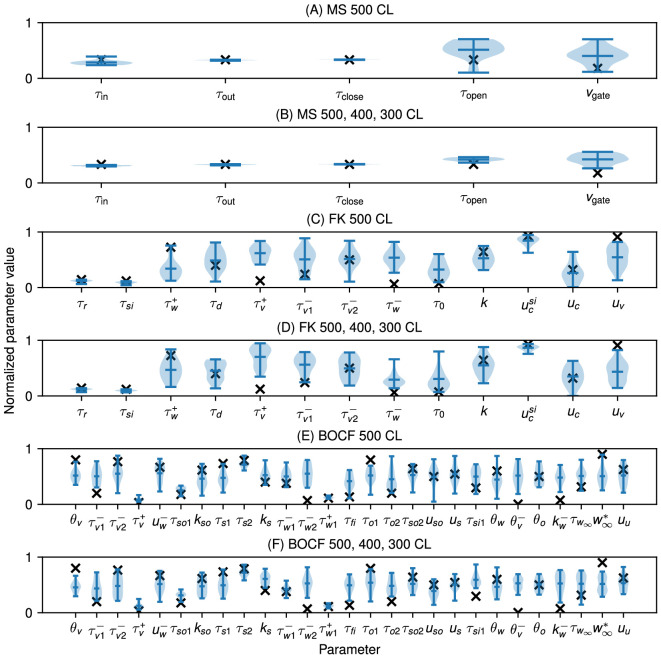
Violin plot of parameter values from the BOCF, FK and MS models fitted to data taken from the respective model itself over 20 runs. The black ‘X’ indicates the value of the parameter used to generate the data. The parameter values are presented normalized over the range of parameter bounds, using the defaults in all cases. (A) MS model fitted to itself, with one dataset with a cycle length of 500 ms. (B) MS model fitted to itself, with three datasets, cycle lengths of 500 ms, 400 ms, 400 ms and 300 ms. (C) FK model fitted to itself, with one dataset with a cycle length of 500 ms. (D) FK model fitted to itself, with three datasets, cycle lengths of 500 ms, 400 ms, 400 ms and 300 ms. (E) BOCF model fitted to itself, with one dataset with a cycle length of 500 ms. (F) BOCF model fitted to itself, with three datasets, cycle lengths of 500 ms, 400 ms, 400 ms and 300 ms. Fits were generated using 4096 particles, 100 iterations and four pre-recording stimuli in all cases.

Because the datasets came from models with known parameter values, these examples also allowed us to examine the accuracy of the parameter values found as well as their variability. Figure 6A,B shows that for the MS model, two of the parameters, $\tau_{\text{close}}$ and $\tau_{\text{out}}$, were identified consistently near the true values across the 20 fittings, while the others showed more variability. The choice of datasets used in the fitting could affect the ability to recover the true parameter values, as shown in figure 6B, where including more cycle lengths resulted in identifiability improvements in the form of reduced standard deviations for $\tau_{\text{in}}$ and, to a lesser degree, for $\tau_{\text{open}}$ and $v_{\text{gate}}$.

Fitting the increased number of parameters for the FK and BOCF models made finding a unique minimizing set of parameter values more challenging, as can be seen in figure 6C–F. In some cases, such as $\tau_{v}^{+}$ for the FK model and $\tau_{w2}^{-}$ for the BOCF model, the range of values obtained for a parameter across the 20 fittings did not even include the original value. Although fitting to more CLs decreased the variability in some of the parameter values obtained using CardioFit (8 of the 13 for the FK model and 18 of the 27 for the BOCF model had lower standard deviations when fit with multiple CLs), it did not guarantee better constraints on all parameters, with many parameter ranges remaining the same and some even increasing (such as k for the FK model and $k_{w}^{-}$ for the BOCF model). This sustained variability in many parameters suggests complex interactions among the parameters along with a limited ability to recover parameters governing the gating variables with only voltage datasets for these models. Nevertheless, a small number of parameters could be identified consistently, including $\tau_{r}$, $\tau_{si}$ and $u_{c}^{si}$ for the FK model and $\tau_{v}^{+}$, $\tau_{so1}$ and $\tau_{w1}^{+}$ for the BOCF model. Such parameters represent values to which the model is highly sensitive. For example, $\tau_{r}$ in the FK model directly governs the strength of the repolarizing current, so that errors in this value noticeably alter the action potential shape.

### 3.2. Fitting to data from other models

**Figure 7.**
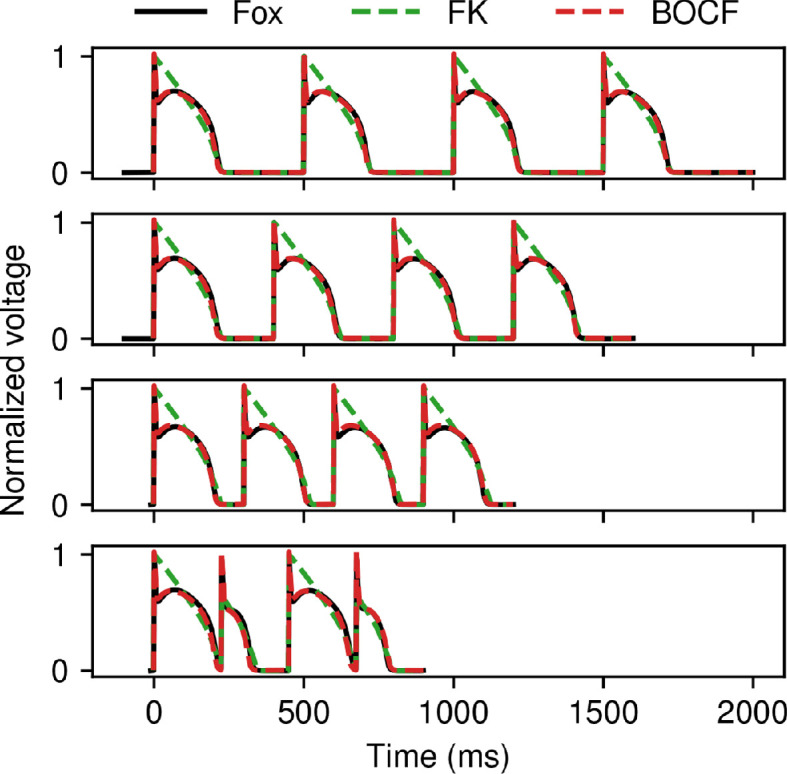
Fitting the FK (green) and BOCF (red) models to normalized voltage data obtained from the Fox *et al.* model (black). Action potentials at CLs of 500 ms, 400 ms, 300 ms and 225 ms were fitted simultaneously to the data shown, with alternans present at the shortest CL. Four pre-recording stimuli, 65 536 particles, and 100 iterations were used in all cases, and the normalization constant was set to 1.4 for the BOCF model.

To demonstrate the versatility of CardioFit, we used it to fit available models to data generated from biophysically detailed models. The resulting parametrizations may allow less computationally expensive models to reproduce aspects of the behaviour of more complex models. Figure 7 shows results from fitting the FK and BOCF models to data from the 13-variable Fox *et al.* canine ventricular AP model [38]. Normalized voltage traces from four different CLs (500, 400, 300 and 225 ms) were fitted simultaneously, with the 225 ms CL producing large-amplitude alternans. As can be seen in figure 7, the FK model (green) could not match the spike-and-dome AP shape of the Fox *et al.* model, but it nevertheless matched the peak voltage value and repolarization phases, while achieving alternans at the shortest CL and avoiding it for the longer CLs. Using the BOCF model (red), the Fox *et al.* data were reproduced more closely over the full APs for all CLs, including during alternans.

**Figure 8.**
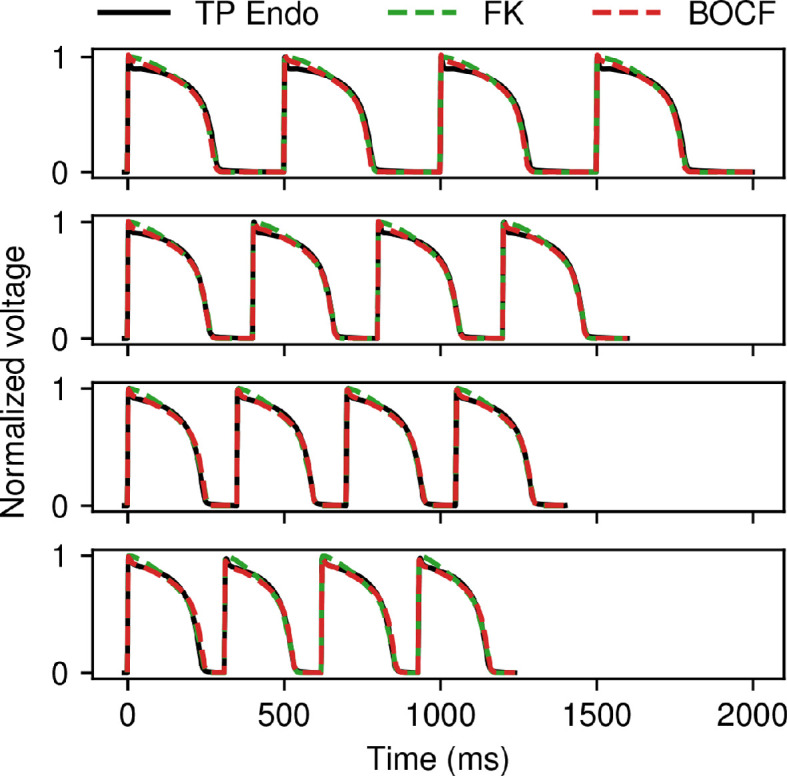
Fitting the FK (green) and BOCF (red) models to normalized voltage data obtained from the ten Tusscher *et al.* model with endocardial tissue parameters (black). Action potentials at CLs of 500 ms, 400 ms, 350 ms and 310 ms were fitted simultaneously to the data shown. Four pre-recording stimuli, 65 536 particles, and 100 iterations were used in all cases, and the normalization constant was set to 1.3 for the BOCF model.

As another example of using CardioFit to fit data from a more complex model, figure 8 shows fits of the FK (green) and BOCF (red) models to normalized endocardial APs from the human ventricular model of ten Tusscher *et al.* [39]. In this case, the four CLs used were 500, 400, 350 and 310 ms, with alternans occurring for the shortest CL. Good agreement was obtained for both the FK and BOCF models with the AP shapes as well as the presence or absence of alternans. The BOCF model more closely fitted the initial repolarization and AP plateau than the FK model due to its greater flexibility in AP shape.

**Figure 9.**
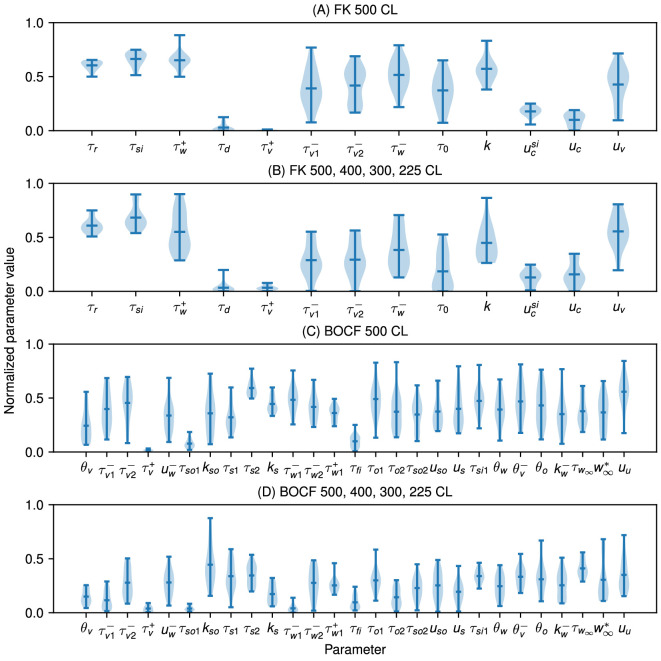
Violin plot of the parameter values of the BOCF and FK models taken over 20 runs using the data from the Fox *et al*. model shown in figure 7. Parameter values are presented as normalized over the range of parameter bounds (the default bounds were used in all cases). (A,B) Parameter values obtained for the FK model fitted (A) to the 500 ms CL data only and (B) to all four CLs. (C,D) Parameter values obtained for the BOCF model fitted (C) to the 500 ms CL data only and (D) to all four CLs. All cases used 4096 particles and 100 iterations, and the normalization constant was set to 1.4 for the BOCF model.

Although there is no true set of parameters for the simplified models in CardioFit when fitting them to data obtained from other models, an examination of the consistency of the parameter values found can reveal the relative importance of the parameters of the simpler models as well as the usefulness of fitting data from multiple CLs. Figure 9 shows the normalized values of each parameter within its default bounds over 20 runs using the data from the Fox *et al.* model described in §3.2 to fit the FK and BOCF models. Although the values for a few parameters for each model occupied only a small portion of the range defined by their bounds, many of the parameters took on values over much of their ranges in parametrizations that gave low-error fits to the voltage data. In general, the FK model parameters seemed more adequately constrained than the parameters of the BOCF model, which are more numerous by about a factor of two. In particular, the values of $\tau_{d}$ and $\tau_{v}^{+}$ were among the most consistent for the FK model. In addition, increasing the number of CLs in the data to be fitted had different effects for the two models, with standard deviations decreasing for only two parameters for the FK model, as shown in figure 9B as compared with figure 9A; for the BOCF model, additional CLs reduced parameter ranges more appreciably, with standard deviations decreasing for 18 parameters, as shown in figure 9D as compared with figure 9C.

### 3.3. Fitting experimental data

**Figure 10.**
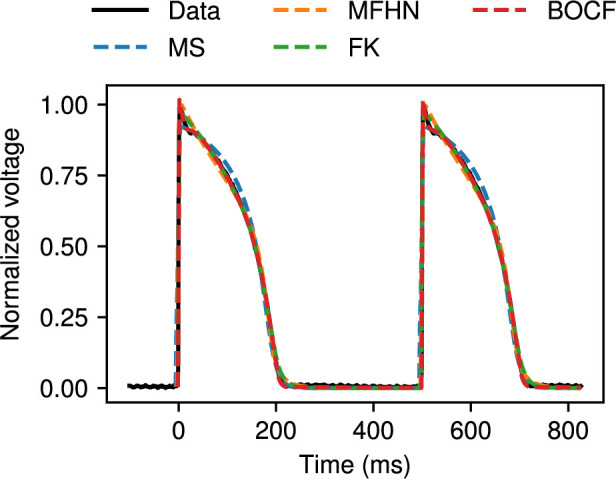
Fitting the MS (blue), MFHN (orange), FK (green) and BOCF (red) models to canine endocardial data paced at 500 ms using 4096 particles, 32 iterations and four pre-recording stimuli, and the normalization constant was set to 1.35 for the BOCF model. Excellent agreement is obtained in all cases, with only small discrepancies shortly after the upstroke.

**Figure 11.**
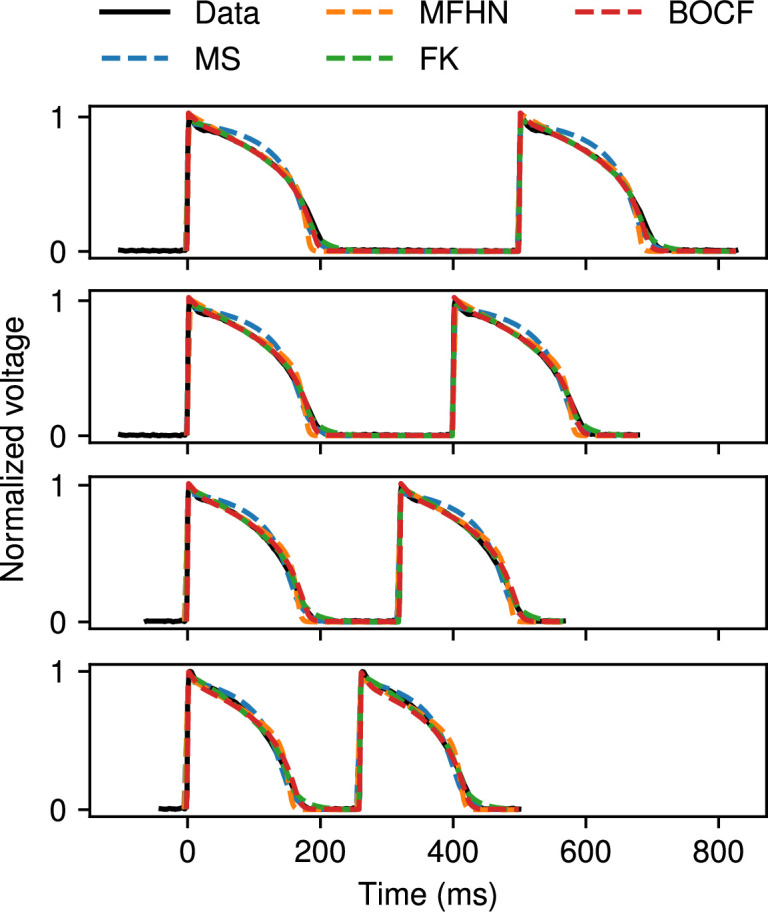
Fitting the MS (blue), MFHN (orange), FK (green) and BOCF (red) models to four sets of canine endocardial data paced at 500 ms, 400 ms, 320 ms and 260 ms, using 4096 particles, 32 iterations and four pre-recording stimuli, and the normalization constant was set to 1.35 for the BOCF model. The FK and BOCF models achieve slightly better fits than the MS model, which tends to produce APs that are less triangular than the APs in this dataset.

We also used CardioFit to fit models to microelectrode recordings of canine endocardial action potentials. Fits of the MS (blue), MFHN (orange), FK (green) and BOCF (red) models to normalized voltage data from a single CL of 500 ms are shown in figure 10. Very good agreement was obtained in all cases, with discrepancies most noticeable at and shortly after reaching the peak voltage value, due to differences in the models’ characteristic action potential shapes. Figure 11 shows the more challenging case of simultaneously fitting data from four different CLs of 500, 400, 320 and 260 ms using the MS (blue), MFHN (orange), FK (green) and BOCF (red) models. In all cases, CardioFit identified a parametrization of the model that closely matched the data within the limitations of the model, with a slightly more noticeable discrepancy for the MS model compared with the other models.

**Figure 12.**
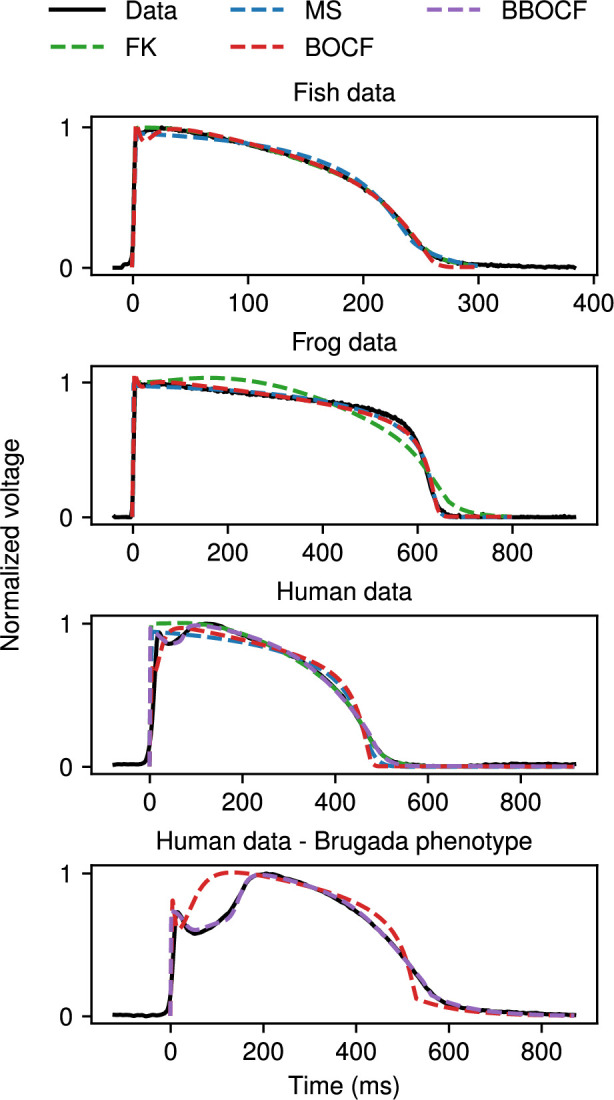
Representative fittings of the MS (blue), FK (green), BOCF (red) and BBOCF (purple) models to experimental data recorded from three different species, including human hearts with and without characteristics of Brugada syndrome. Note that for the last case (human data with Brugada phenotype), only BOCF and BBOCF model fits are shown. All cases use 1024 particles and 32 iterations with no pre-recording stimuli except for the last case, which uses 16 384 particles and 70 iterations, with the upper bounds for $\tau_{o1}$ and $\tau_{o2}$ increased to 1000 and 200, respectively, for both models to accommodate the slow recovery time. The normalization constant was set to 1 for the BOCF model for the human data with Brugada phenotype, and to 1.05 and 1.35 for the BBOCF model for the normal and Brugada human cases, respectively.

As further examples, figure 12 shows fittings of the MS (blue), FK (green) and BOCF (red) models to ventricular APs recorded from three different species, zebrafish, frog and human, including a fairly typical AP and one showing prolongation with a shape characteristic of Brugada syndrome. While the frog and fish data were recorded via microelectrode (paced at 800 ms and 300 ms, respectively), the human data were taken from an optical-mapping experiment performed on an excised human heart (paced at 1000 ms). Despite these data showing a wide variance in both their APDs and AP shapes, CardioFit found low-error parametrizations for the FK, MS and BOCF models, with the frog AP shape proving difficult for the FK model to match. In addition, the BBOCF model (purple) was also used to fit the human tissue data, resulting in a better reproduction of the more complex morphology present in the human dataset compared with the other models. For the Brugada phenotype, only the BBOCF model was capable of matching the ‘saddleback’ shape; for comparison, figure 12 also includes a fitting of this dataset obtained using the BOCF model.

Our fittings indicate key parameters involved in fitting action potentials from the four species (canine, fish, frog and human) to the different models. In particular, we tested whether the parameter values obtained from 20 runs of CardioFit produced different parameter means and standard deviations for all six pairwise comparisons of the four species. For the MS model, $\tau_{\text{in}}$ (upstroke time scale) and $\tau_{\text{close}}$ (plateau time scale) were different for all pairwise comparisons; in fact, the only species pairs with parameter values that were not different were the frog and canine ($\tau_{\text{open}}$ and $v_{\text{gate}}$) and the fish and frog ($\tau_{\text{out}}$ and $v_{\text{gate}}$). For the FK model, there were no individual parameters whose values were different across all species comparisons, but parameters that were most commonly fitted to different values across the species included $\tau_{r}$, $\tau_{w}^{+}$, $\tau_{w}^{-}$, $u_{c}$ and $u_{c}^{si}$. For the BOCF model, the parameters most commonly involved in differentiating the action potentials for different species included $\theta_{v}$, $k_{so}$, $\tau_{s1}$, $\tau_{s2}$, $\tau_{w1}^{+}$, $\tau_{fi}$ and $u_{u}$. Details are given in electronic supplementary material, tables S9–S11.

In addition, our results using the BBOCF model, which obtained high-quality fittings for both the normal and Brugada-phenotype action potentials, indicated that several parameters play key roles in changing from the normal to Brugada-type shape. Specifically, the values obtained across 20 fittings for each case achieved different means for the following parameters (p<0.001): $\tau_{w2}^{+}$, $\tau_{s2}$, $\tau_{o2}$, $\tau_{si1}$, $\tau_{si2}$, $k_{so}$, $s_{c}$ and $u_{so}$. The parameter $k_{si}$ was also different for the two datasets at the level p<0.01. More description of the roles of these parameters can be found in §4.

### 3.4. Fitting with action potential duration data

**Figure 13.**
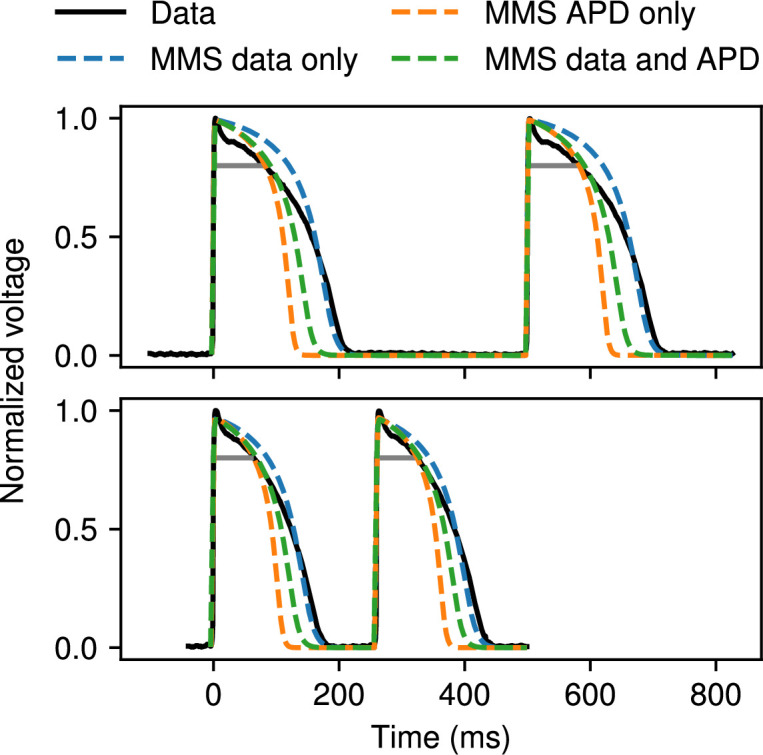
Fitting of the MMS model to canine endocardial data of cycle lengths 500 ms and 260 ms. The results of fitting to the voltage data only (blue), fitting to APD data only (orange), and a simultaneous fitting of voltage and APD data minimizing the APD error (green) are shown. The grey lines indicate the APD of the voltage data taken at a voltage value of 0.8. All cases used 1024 particles and 32 iterations of PSO. Four pre-recording stimuli were used in all cases.

When matching the APD of a dataset is particularly important, the APD fitting feature of CardioFit can be used. This feature is particularly important for the two-variable models, which otherwise do not emphasize APD accuracy and may achieve low curve error overall while having higher error during repolarization. An example of a fitting to both voltage and APD data is shown in figure 13, where the MMS model was fitted to canine endocardial data at CLs of 500 ms and 260 ms; APDs for this case were measured using a threshold of 0.8 corresponding to ${\text{APD}}_{20}$, which facilitates demonstration of the APD fitting feature. Fitting to voltage alone resulted in ${\text{APD}}_{20}$ errors of 46% and 35% for the 500 ms and 260 ms cases, respectively. Fitting to the APD only resulted in respective APD errors of 0% and 1%, whereas the hybrid fitting resulted in respective APD errors of 10% and 8%. By simultaneously fitting the APD and the voltage data, a more accurate APD fitting was achieved than by fitting voltage alone at the cost of slightly higher curve error. The hybrid case used fitting weights of 1000 for the APD data and 0.1 for the voltage trace. CardioFit also allows the option to fit APDs without using any voltage data, resulting in the highest curve error, but giving more flexibility when limited data are available, such as for clinical data.

Performing parameter fittings using the more limited data provided by APDs only, without accompanying voltage traces, would be expected to affect the variability of fitted parameter values. To assess robustness when fitting only APD, we considered 20 repeated fittings of the MS, FK and BOCF models using only APD data derived from the same model. For the MS model, the voltage trace was well recovered from the APD data alone, although with slightly more variation than fitting to the voltage time series directly (see electronic supplementary material, figure S3 compared with figure 3). The likely explanation for the similarity to the original data is the relatively prescribed action potential shape of the MS model given an APD value, as the small number of variables and parameters limit the model’s flexibility. For the FK model, the fittings obtained were able to match the APD data, but, in contrast to fitting to the time series directly, none of the 20 fits accurately recovered the voltage time series behaviour (see electronic supplementary material, figure S4 compared with figure 4). The fittings of the BOCF model to APD data also reproduced the APD values but produced a wide variety of AP plateau behaviour, although this property was well constrained when fitting to the full time series data (see electronic supplementary material, figure S5 compared with figure 5).

Violin plots of the parameter distributions for the APD self-fitting cases, shown in electronic supplementary material, figure S6, indicate that the parameters of the models generally were not well constrained by the APD data alone, in contrast with the parameter distributions for the same fittings to time series data, shown in figure 6, where several parameters were constrained for each model even when fitting to only a single cycle length. The only exception was the MS model, where the value of $\tau_{\text{open}}$ was similarly constrained as with the time-series fit (figure 6B and electronic supplementary material, figure S6B).

### 3.5. Scaling and convergence

**Figure 14.**
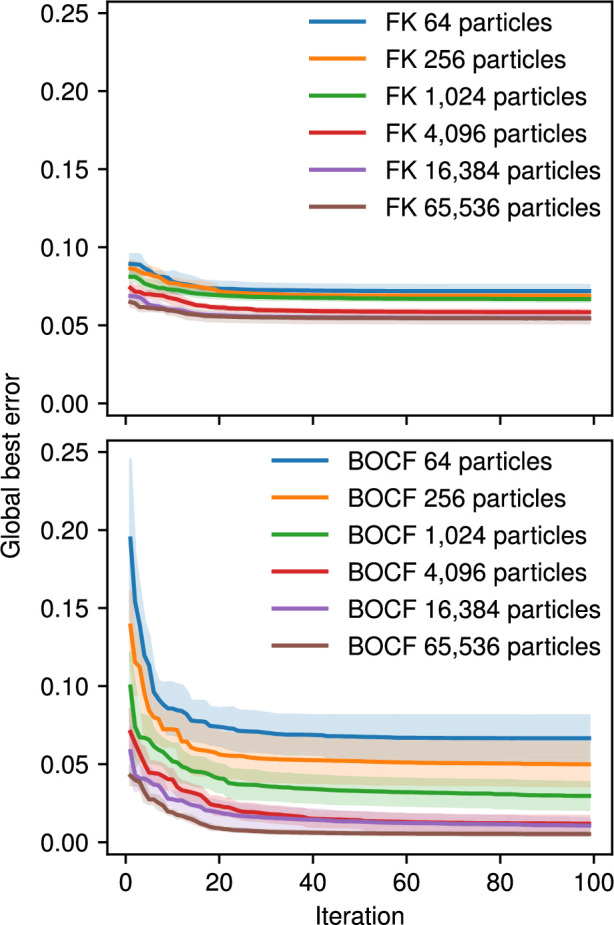
Global best error for each iteration averaged over five CardioFit runs. The shaded ribbons indicate the standard deviation of the error at that iteration over the five runs. Fits used the data from the four CLs of the Fox *et al.* model shown in figure 7. The PSO algorithm was run for 1000 iterations, but only the first 100 are shown, as very little improvement occurred after this point.

One of the advantages of using graphics hardware for parallelization is that CardioFit can use a large number of particles for the PSO algorithm. Increasing the number of particles generally leads to a final result with lower error, although individual cases do not always follow this trend due to the non-deterministic nature of the algorithm. We observed that increasing the number of particles was generally the most effective way to reduce error when fitting a variety of datasets and models. Examples of the error convergence behaviour of CardioFit using the Fox *et al.* model dataset fitted to the FK and BOCF models are shown in figure 14.

Because the algorithm is guaranteed to converge within a finite number of iterations (see §2.3), increasing the number of iterations does not necessarily improve the result. However, the number of iterations required for convergence varies depending on the model, number of particles and data being fitted. When using CardioFit, the convergence plot shown at the bottom of the page (see electronic supplementary material, figure S2) can serve as a useful indicator of whether more iterations would improve the fit. In the cases shown in figure 14, increasing the number of iterations did not compensate for using fewer particles. Although the error improved in a few cases after many iterations, the improvement was slight relative to the improvement resulting from increasing the number of particles. Figure 14 also demonstrates that the best error achieved (depicted by the shaded regions) for a given choice of model, number of particles and number of iterations could have significant variance, a consequence of the non-determinism inherent in the PSO algorithm.

### 3.6. Program execution time

One of the primary goals of CardioFit is to produce results quickly—ideally in a matter of seconds—without sacrificing the quality of the fitting. Several factors affect the running time of CardioFit beyond the implementation. Each iteration will take more time if more simulation time is required to compare the model output with the data. Consequently, increasing the number of data files, number of applied stimuli and pre-recording stimuli, number of cycle lengths or complexity of the model to be fitted generally will increase the running time. Additionally, increasing the number of iterations will increase the running time, with each iteration taking a relatively consistent amount of time given the other factors. Increasing the number of particles also may increase the running time, although the exact relationship between number of particles and time depends on the specific hardware and drivers used to run CardioFit.

**Figure 15.**
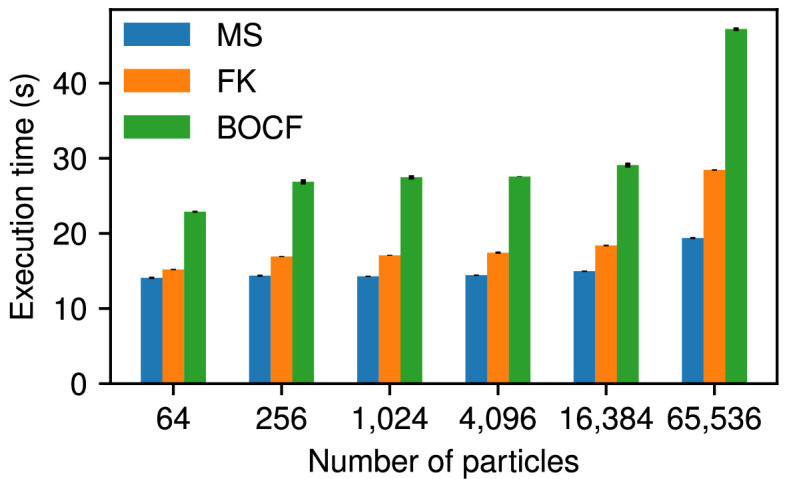
Average time over five runs of CardioFit to fit various models to the data shown in figure 7 for varying numbers of particles. Each run used 100 iterations of the algorithm with four pre-recording stimuli and four stimuli applied. The standard deviation of the execution times is shown by the black line at the top of each bar.

To demonstrate the speed of the program, as well as the effects of varying the number of particles, timing results are presented in figure 15 for fitting to the Fox *et al.* model data described in §3.2 with four stimuli and four pre-recording stimuli. Results were recorded on a computer using an AMD Ryzen 9 7950X with 16 cores (32 virtual) with a clock speed of 5.881 GHz, an AMD RX 7900 XTX GPU and 64 GB RAM, running the Firefox Web browser. In this case, the number of iterations was held constant at 100 iterations while the model and number of particles were varied. Even the slowest case attempted, the BOCF model with 65 536 particles, completed an average run in less than 50 s. Model choice significantly impacts execution time; the BOCF model requires approximately double the time of the much simpler MS model in many cases, with the FK model lying between the two. The number of particles also plays a significant role, although the cost of a few seconds required to use 4096 particles as opposed to 64 particles may be worth the higher-quality fits produced by more particles, especially when most modern graphics cards can comfortably accommodate thousands of particles.

The fact that the running time does not increase proportionately to the number of particles is explained by the extreme parallelism offered by GPUs. In many cases, the GPU may have enough processing power that reducing the number of particles simply leaves available processing power idle, which does not lead to speedup. Once the number of particles is large enough that the GPU must work at full capacity, CardioFit will take longer to run as the number of particles is increased further. However, the exact speeds and behaviour will depend on many factors, most notably the specific processor, graphics card, drivers and browser version of the computer running the program.

## 4. Discussion

CardioFit allows fast and easy fitting of a selection of cardiac models to both voltage time series and APD data, which we have found to be successful for matching the output of both other models and experimental data. We note in particular that users do not need to write or modify any code to obtain fits from the tool, and all fits shown were obtained within a matter of seconds, even on laptops without dedicated graphics hardware. In addition, CardioFit does not require any machine-specific compilation or optimization, and may be run in a Web browser simply by loading the Web page. Where possible, CardioFit provides end-users with sensible defaults for the optimization hyperparameters and model constraints to minimize the effort required to produce a model fit. Nevertheless, CardioFit users retain the ability to control the hyperparameters of the PSO algorithm and constraints on the model through an interactive user interface. Thus, CardioFit provides an experience that can meet the needs of both new and experienced users. In addition, the fittings that CardioFit obtains can provide insights into the roles of model parameters as well as the identifiability of the model given the provided data.

### 4.1. Insights into model parameter roles

Our study helps to identify the parameters of individual models that support differentiating action potentials across species. For the MS model, which has a small number of parameters whose roles tend to be fairly specific and narrowly defined, most parameter values were found to be different across pairwise species comparisons (see electronic supplementary material, table S9). All parameter values obtained were different for the human dataset compared with the canine, fish and frog data, and between the canine and fish datasets. Cases where values were not found to be different ($\tau_{\text{open}}$ and $v_{\text{gate}}$ for canine versus frog and $\tau_{\text{out}}$ and $v_{\text{gate}}$ for fish versus frog) could indicate either that the values were the same or that the variability was too high to make a meaningful distinction.

For the FK model, more variability in the parameters that were different was observed in pairwise species comparisons. The most commonly different parameter values were $\tau_{r}$, which governs the strength of the repolarizing current; $\tau_{w}^{+}$ and $\tau_{w}^{-}$, which set the time constant of the slow inward current’s gating variable and thus are primary contributors to action potential duration; and the threshold values $u_{c}$ and $u_{c}^{\text{si}}$. Interestingly, only three parameters were reliably distinguished between the canine and human datasets: $\tau_{w}^{-}$; $\tau_{v1}^{-}$, which affects how the fast inward current turns off; and k, which affects the slow inward current formation. Two parameters were found never to have meaningfully different values: $\tau_{v2}^{-}$ and $u_{v}$, which are both involved in the late stages of recovery of the v gate and likely could not be pinpointed from the limited datasets.

The BOCF model similarly tended to find differences when comparing the fittings pairwise across species for variables affecting repolarizing current strength (e.g. $\tau_{s1}$, $\tau_{s2}$ and $k_{\text{so}}$), the fast inward current ($\tau_{\text{fi}}$, $\theta_{v}$ and $u_{u}$), and $\tau_{w1}^{+}$. Both the fish versus canine and fish versus frog comparisons had few meaningfully different values, whereas the human versus canine comparison had statistically significantly different values for all but three parameters. As with the FK model, $\tau_{v2}^{-}$ was never found to be different across the pairwise species comparisons.

Further insights can be obtained by comparing the BBOCF model parameter values obtained by fitting the normal and Brugada-type human action potentials. Of the nine parameters found to have statistically significantly different values for the two cases, four ($\tau_{\text{si}1}$, $\tau_{\text{si}2}$, $s_{c}$ and $k_{\text{si}}$) are used to define the time constant of the slow inward current (which is inversely proportional to the current’s magnitude). The change in the magnitude of this current is consistent with the need to prolong the action potential for the Brugada phenotype. Parameters $k_{\text{so}}$ and $u_{\text{so}}$ affect the sigmoidal transition between the two time constants that affect the magnitude of the slow outward current for larger voltage values, thereby helping to form the saddleback feature. The remaining parameters affect the recovery of the activation and inactivation gating variables for the slow inward current, which also helps to prolong the action potential, as well as the magnitude of the slow outward current during the beginning of the late repolarization phase. Those parameters whose values were not statistically significant can be considered not important in producing the Brugada-phenotype action potential.

### 4.2. Insights into model structure and parameter identifiability

As can be seen in figures 6 and 9, we found substantial variability in the values of a number of model parameters across repeated fittings to the same data, even though the voltage traces from the different parametrizations fit the data well (see figures 3–5). Lower variability typically was seen for some parameters of the MS model; for the FK and BOCF models, low variability generally was seen for some parameters, but the specific parameters with low variability depended on the dataset. Including more data through additional cycle lengths tended to decrease the variability in at least some parameters. Such behaviour was enhanced when fitting APD values alone; compare the increased variability of the violin plots in electronic supplementary material, figure S6 when fitting APD only with those in figure 6.

The variability in parameter values is related to parameter identifiability, an area of ongoing research for cardiac models [42,43], and may arise from a number of different model or data features. For example, there may not be sufficient data to constrain certain values, such as parameters associated with the minimum diastolic interval or conduction velocity properties (although the biphasic stimulus protocol can help to avoid at least some parameter combinations that would fail to propagate in tissue [14]). A specific example of such a case can be seen in figure 6A,B, which show the most variability for MS model parameters $\tau_{\text{open}}$ and $v_{\text{gate}}$. Given that the data used for fitting consisted of voltage traces at pacing rates far from the minimum diastolic interval, difficulty in estimating $\tau_{\text{open}}$, which governs the minimum diastolic interval, and $v_{\text{gate}}$, which specifies the threshold for excitation, aligns with expectations. Furthermore, a model may be limited by its structure and thus may not accommodate particular AP shapes, leading to irregular outcomes. In addition, the model may be insensitive to a particular parameter, or it may be possible for the model to compensate for an error in one parameter with an error in one or more other parameters within the context of the data to which the model is being fitted. Fitting to different types of data, such as data generated from a stochastic pacing protocol [44], may be a valuable approach towards reducing variability in parameter values.

The limitations imposed by identifiability challenges associated with particular choices of models and data to fitted suggest practical uses for CardioFit. Although phenomenological models do not have the same types of physiologically meaningful parameters as biophysically based models, many parameters can be understood as key determinants of particular quantities (e.g. threshold of excitability, minimum diastolic interval, maximum APD, restitution curve slope, etc.). While not required to use CardioFit, user knowledge of the roles of model parameters can help in setting expectations regarding identifiability and in choosing a subset of parameters to fit that that will be most relevant to a specific question of interest. In the absence of a thorough understanding of the roles of some parameters (which seems likely in models with more parameters, like the BOCF and BBOCF models), performing multiple runs such those whose results are depicted in figures 6 and 9 may be useful in determining a small subset of parameters to which the model is most sensitive and thus whose values are most likely to be constrained by the data provided. Users can then fix the values of the remaining parameters based on established parameter sets for the model and focus on fitting the subset of selected parameters. Within the specific context of use [45], users can test the sensitivity of predictions to the unfit parameters either by using conventional means (e.g. varying each parameter value by a fixed percentage and quantifying the effect) or by using multiple fittings (similar to a population of models [7]) to examine whether a particular conclusion depends on the precise values of parameters that could not be identified reliably from the fitting.

### 4.3. Limitations and future work

A number of modifications or extensions could be made to CardioFit that could lead to improved performance or additional functionality. Currently, CardioFit offers the ability to fit data to six phenomenological models. However, allowing fittings to more detailed models, especially popular models of human ventricular cells [39,46], would increase CardioFit’s usefulness. We intend to add these models to a future version of CardioFit, with the ability to fit maximal conductances of currents and possibly scale factors for gating variable time constants.

Our choice of PSO was motivated by certain advantages it provides: for example, adding a model requires only its implementation as a simulation in WebGL shader code without a detailed analysis or transformation for the optimization problem, unlike linear regression or quadratic optimization approaches. In addition, PSO is capable of attaining rapid repeated fits on most hardware due to its efficient parallelization of the PSO algorithm. Nevertheless, it would be possible to use a different fitting algorithm in addition to or instead of PSO. Although the speedup provided by parallelization allows enough iterations and particles to be used in the PSO process that we did not find it necessary to use further optimization methods on the result, as has been done previously [21], it would be possible to include a local optimization as a final step. Other approaches could be explored as well, including genetic algorithms [13,14] and Bayesian approaches [25,30].

As the behaviour of the PSO algorithm is closely coupled to the error metric used to evaluate the particles, one potential improvement to our software would be to support different error metrics. For example, certain parts of the voltage time series (e.g. the AP upstroke) could be weighted more heavily than others, thereby emphasizing specific lower-level features of the data beyond what the fitting weight parameters currently provided at the dataset level can support. Error metrics other than squared error may also be beneficial; for instance, it has been suggested that the coefficient of determination may be a more appropriate metric for regression problems [47]. The simple curve error currently utilized also may also lead to problems when data are not representative of correct model behaviour. For instance, optical-mapping data typically contain a slow upstroke due to spatial averaging, which is not behaviour models should attempt to reproduce. Currently, addressing upstroke blunting in optical-mapping data would require a separate tuning stage for upstroke parameters, whose values could then be held constant while other parameters were varied, but it is possible that greater improvement could be seen by excluding the upstroke from the error metric on the later fitting stages. We also note that error measured during alternans could be artificially high due to a mismatch of long and short APs between the data and the fitting. At present such a misalignment can be compensated globally by increasing or decreasing the number of pre-recording stimuli by one. Approaches to ensure long APs and short APs are compared directly, such as by calculating error with both options and using the lower value, could be considered for a future update of CardioFit.

Additional potential improvements could be made to the data and protocols that CardioFit supports. For example, an option to include a simultaneous calcium transient recording could be added, which could lead to improved fittings. The normalization constant could be included as a hyperparameter whose value is determined along with the model parameters as part of the optimization process. Data obtained from protocols other than constant pacing at one or more fixed CLs, such as an irregular pacing protocol [15], could be allowed and may present novel features that better constrain parameters. Additional stimulus shapes could be incorporated beyond the square pulse and biphasic options. Another current limitation is that reaching short CLs without developing block may be difficult currently because the same initial conditions are used for each CL fit, but CardioFit could be modified to allow the final conditions of one CL to be used as initial conditions for the next shorter CL.

A significant future challenge is to consider tissue simulations, as models often behave differently in simulated tissue when compared with single-cell simulations [2,48]. Such a change would require a significant increase in computation and in the complexity of CardioFit and would require careful consideration to avoid hindering CardioFit’s speed and simplicity from the end-user’s perspective. Finally, because CardioFit generates many sets of parameters that fit datasets relatively well, it could be extended in a different direction through modification to support the generation of populations of models [7] or parameter distributions [26,30].

## 5. Conclusion

We present CardioFit, a fast, user-friendly tool for identifying parametrizations of simple cardiac models to closely match a set of input data. All model parameters, or any selected subset of parameters, may be fitted simultaneously, and a single parametrization may be generated to simultaneously fit multiple datasets taken from different pacing rates. The speed and power of this software are made possible by the capabilities of shader programs to take advantage of parallel graphics hardware available on almost all modern consumer-grade computers. The fittings obtained can promote new insights into the roles of model parameters and the identifiability of models given specific data. Overall, we believe that CardioFit represents an important step towards making flexible and efficient phenomenological cardiac models practical and easy to use in patient-specific applications for modelling experts and non-experts.

## Data Availability

Data and relevant code for this research work are stored in GitHub [49] and have been archived within the Zenodo repository [50]. Electronic supplementary material is available online [51].

## Footnotes

^1^
https://github.com/mcomstock/cardiofit-royalsociety-2025.

^2^
https://mcomstock.github.io/cardiofit-royalsociety-2025.

^3^
https://github.com/mcomstock/cardiofit-royalsociety-2025/tree/master/sample-data.

^4^
https://github.com/mcomstock/cardiofit-royalsociety-2025/blob/master/README.md.
